# Potential quorum-sensing inhibitor of *Hafnia alvei* H4—theaflavin-3,3´-digallate analyzed by virtual screening and molecular simulation

**DOI:** 10.1128/spectrum.02671-23

**Published:** 2023-09-21

**Authors:** Xue Li, Congyang Yan, Yanan Wang, Gongliang Zhang, Jingran Bi, Hongshun Hao, Hongman Hou

**Affiliations:** 1 School of Food Science and Technology, Dalian Polytechnic University, Dalian, China; 2 Liaoning Key Lab for Aquatic Processing Quality and Safety, Dalian, China; University of Minnesota Twin Cities, St. Paul, Minnesota, USA

**Keywords:** quorum sensing inhibitor, virtual screening, TF3, *Hafnia alvei*, molecular docking, molecular dynamics simulation

## Abstract

*Hafnia* species can cause food spoilage via the quorum-sensing (QS) system. Thus, strategies that target QS in these bacteria might be a good approach to safeguard the quality of processed food. In this study, the amino acid sequence of the LasI*_Ha_* protein, a key QS regulator from *Hafnia alvei* H4, was used to construct its 3D structure for the virtual screening of potential QS inhibitors (QSIs) from the Bioactive Compound database. Four potential QSIs were obtained, and these were all theaflavins (TFs). Among them, theaflavin-3,3´-digallate (TF3) was found to outperform the others, displaying a higher docking score according to molecular docking analysis, and required only a sub-minimal inhibitory concentration (31.25 mM) to cause a significant decrease in the production of the autoinducer N-acyl homoserine lactone in *H. alvei* H4 and up to 60.5% inhibition of its motility. Furthermore, molecular simulation results indicated that TF3 could stably bind to a cavity within LasI*_Ha_* to form stable hydrogen bonds and hydrophobic interactions with various key residues of the protein to exert the inhibitory effect. Thus, TF3 may be considered a potential compound to protect against food spoilage caused by *H. alvei* H4 via the quorum quenching.

**IMPORTANCE:**

*Hafnia alvei*, the main strain studied in this paper, is often isolated from spoiled foods, especially refrigerated protein-based raw foods, and is generally considered to be a spoilage bacterium whose spoilage-causing properties may be closely related to its own very strong population-sensing activity, so the strategy of quorum quenching against *H. alvei* H4 may be a good way to guarantee the quality of processed foods. Given the current global requirements for food safety and quality, coupled with negative consumer perceptions of the excessive inclusion of synthetic chemicals in food products, the use of natural compounds as QSIs in the storage of aquatic food products would seem more attractive.

## INTRODUCTION

Quorum sensing (QS) is the “bacterial language” using chemical molecules known as autoinducers (AIs) generated during cell proliferation to facilitate cell-cell communication (1). The most prevalent family of AIs employed by Gram-negative proteobacteria is N-acyl homoserine lactone (AHL), which is synthesized by AHL-synthases (a LuxI-type family protein) using S-adenosyl-L-methionine and acyl-acyl-carrier protein (acyl-ACP) as substrates. When the concentration of AHL reaches a threshold as a result of an increase in bacterial cell density, members of the LuxR protein family would bind to the AHL to activate the expression of downstream genes to control a vast set of relevant phenotypes, including cell motility, biofilm formation, and virulence factors (2, 3). Previous research has shown that QS plays an important role in the bacteria-mediated deterioration of seafood such as giant yellow croakers (4). In addition, the QS system may also work in conjunction with other bacterial products to bring about food spoilage, and these products include bacterial proteases, lipases, and biofilms. One example of a food spoilage bacterial species is *Hafnia alvei*, which is an important chilling spoilage bacterium often found in decaying food. Many studies have highlighted the crucial role of the QS system-controlled virulence factors and biofilm production of *H. alvei* in food spoilage (5, 6). Tan et al. (7) isolated *H. alvei* from spherical fish and detected two short-chain AHLs by lLiquid chromatograph-mass spectrometer (LC-MS): 3-oxo-C6-HSL and 3-oxo-C8-HSL. Christensen et al. (8) showed that the specific spoilage bacteria of rainbow trout were *H. alvei*, *Serratia liquefaciens*, and *Pseudomonas fluorescens*, which produced AHLs such as 3-oxo-C6-HSL and C6-HSL that were able to regulate protein hydrolase activity and spoilage of rainbow trout fillets. In addition, some studies found that the specific spoilage organisms (SSOs) in turbot under 4℃ refrigerated condition were *P. fluorescens* and *H. alvei* (9). In addition, although the number of *H. alvei* was low in aquatic products, the growth of other non-cold-tolerant bacteria was inhibited when low temperature conditions were reached, while *H. alvei* could become the SSO under low temperature conditions and its putrefactive ability is regulated by the QS system (9). Thus, focusing on the bacterial QS system might be a potential tactic to delay the onset of bacterial-mediated food spoilage.

Bacterial putrefaction can be avoided by natural or deliberate interference that inhibits or disrupts QS. QS inhibitors (QSIs) are chemicals that do not affect the growth of the bacteria but only block, interfere with, or inhibit the exchange of information among the bacterial cells by affecting the QS system. The best QSI is one that can be used at a concentration that is non-toxic to humans and has little impact on the growth of the bacterial cells in terms of direct killing while displaying maximum inhibition of QS activity. QSIs such as quercetin and L-carvone (10) have been found to interfere with QS primarily by competing with AHL for binding with the receptor to inhibit the expression of downstream genes involved in various processes, such as bioluminescence and pigment or antibiotic production (11). Another approach to interfere with the QS system is to directly inhibit the synthesis of AHLs by targeting specific AHL synthase. Such an approach would require a better understanding of the AHL synthesis process and the mechanisms of these molecules from the structural perspective. So far, the 3D structure of LasI except for the LasI*_Ps_* from *Pseudomonas aeruginosa* has not been determined (12). LasI*_Ps_* has less restriction on the length of the acyl chain in AHLs, as it can accommodate AHLs with an acyl chain ranging from C4 to C8. In addition, a structural study of EsaI from *Pantoea stewartii* has identified a hydrophobic pocket within the protein for binding the acyl chain (13, 14). Since traditional methods of screening QSIs are time consuming and inefficient, a virtual screening technique has been introduced to reduce screening time. This process is based on molecular docking that involves performing a large number of rapid screenings of compounds in a small molecule library to achieve shape and energy matches between ligands and receptors (15). Although the 3D structure of *H. alvei* H4 LasI*_Ha_* has not been reported, the availability of its protein sequence and the 3D structures of *P. aeruginosa* LasI*_Ps_* and *P. stewartii* EsaI could provide sufficient information for constructing the 3D structure of *H. alvei* H4 LasI*_Ha_* via homology modeling.

In this study, we screened approximately 50,000 small molecule compounds from the MCE Bioactive Compound Library (https://www.medchemexpress.com/screening/BioactiveCompoundLibrary.html) for potential QSIs against the LasI*_Ha_* protein of *H. alvei* H4. One theaflavin-type (TF) compound, theaflavin-3,3´-digallate (TF3), was found to exhibit the strongest QS inhibitory activity and the lowest minimum inhibitory concentration (MIC) value. Phenotypic studies showed that TF3 could remarkably reduce the production of AHLs and significantly inhibit the motility, biofilm formation, and expression of QS-related genes in these strains. In addition, the result of molecular simulation further validated the QS inhibitory effect of TF3. Given the current global requirements for food safety and quality, coupled with negative consumer perceptions of the excessive inclusion of synthetic chemicals in food products, the use of natural compounds as QSIs in the storage of aquatic food products would seem more attractive.

## MATERIALS AND METHODS

### Bacterial strains, chemicals, and culture conditions

The bacterial strains in this study were wild-type (WT) *H. alvei* H4 and two mutants previously constructed in our laboratory (16), one of which lacks the *lasI* gene (Δ*lasI*), whereas the other lacks the *expR* gene (Δ*expR*). Both wild-type and mutants were cultured in Luria-Bertani (LB) broth medium at 30°C with shaking at 150 rpm. In addition, the mini-Tn5 mutant of *Chromobacterium violaceum* (CV026) provided by the Chinese Academy of Agricultural Sciences (17) was also used in this study. The QS signaling molecule C6-HSL was purchased from Sigma Aldrich (St. Louis, MO, USA). All small molecule compounds for potential QSIs validation assay were purchased from MedChemExpress (MCE, Shanghai, China) with a purity of 98%. Each compound was dissolved in sterile water or dimethyl sulfoxide (DMSO). Thirty percent DMSO and sterile water were used as controls.

### Protein and ligand preparation

The amino acid sequence of *H. alvei* H4 LasI*_Ha_* was taken from our previous publication (18). The sequence was then submitted to SWISS-MODEL (https://swissmodel.expasy.org) (19) to construct a 3D structure. The best model with the highest similarity and GMQE (Global Model Quality Evaluation) scores was selected from the 50 models built by SWISS-MODEL. The chosen 3D structure was evaluated by using the SWISS-MODEL and Ramachandran Plot. To determine the extent of protein sequence similarity between *H. alvei* H4 LasI*_Ha_* and related proteins from other species, its amino acid sequence was aligned with the sequences of the most frequently studied AHL synthases, LuxI, RhlI, and EsaI as well as the *P. aeruginosa* LasI*_Ps_* using CLUSTALW (https://www.genome.jp/tools-bin/clustalw), and the final alignment result was displayed by ESPript 3 (https://espript.ibcp.fr/ESPript/ESPript/index.php).

The LasI*_Ha_* protein was then subjected to further fine-tuning, such as hydrogenation, removal of water molecules, and repair of missing residues and side chains due to missing residues in the template side chain using the Protein Preparation Wizard module. Subsequently, energy optimization (OPLS2005 force field, root mean square deviation [RMSD] = 0.30 Å) was performed. The final version of the protein was used to make lattice files with the Receptor Grid Generation module for subsequent docking. All other parameters were set to default.

The 2D format of MCE Bioactive Compound Library was processed by hydrogenation and energy optimization through the LigPrep Module of Schrödinger software 11.4 (LLC, NY, USA, 2018-4), and the 3D structure was output for virtual screening. MCE Bioactive Compound Library contains ~50 k compounds distributed in the natural product library, drug and food homology library, food-derived component library, and food additive library.

### Virtual screening

Structure-based virtual screening against approximately 50,000 compounds was performed by Schrödinger Maestro 11.4 software to identify potent molecules that could interact with the active pocket of LasI*_Ha_*. The Virtual Screening Workflow module was used for the screening process. After the import of the 50,000 compounds, molecular docking was performed with the Glide module to examine the docking between receptor and ligand molecules based on geometric and energy matching. First, the compounds in MCE Bioactive Compound Library were screened by the high-throughput screening mode of the Glide module, and then the top 30% of the small molecule compounds were selected by the standard (SP) mode for the second round of screening. Next, the compounds from the top 30% score were selected for the third round of screening with high precision (XP) mode to obtain a ranking for the compounds. The binding ability of each compound and the structures of the protein and compounds were manually reviewed to select the top 200 output compounds from MCE Bioactive Compound Library. After completing the virtual screen, a total of 11 compounds that yielded high docking scores ( >8) or normally found as food components were selected from the top 200 for further experimental validation.

### Identification of anti-QS activity

Anti-QS activity assay was conducted according to a previously described method (20). A sample of CV026 suspension was mixed with nutrient agar medium at a cell-to-medium ratio of 1:50. This was followed by the addition of 20 mg/mL C6-HSL, and the mixture was then poured onto a plate. After solidification, wells were punched in the agar with Oxford cups. Subsequently, 60 µL of a test compound was added per well. Anti-QS activity was assessed by the presence of a colorless, opaque but turbid halo around the well.

### MIC assay and growth curves

A series of twofold dilutions was prepared for each compound in a 96-well plate to give a final concentration range from 0.03 to 2.0 mM for CV026 and 3–600 µM for three *H*. *alvei* H4 strains. A suspension of an overnight bacterial culture was added to each well containing a test compound and then incubated for 24 h. The MIC value was defined as the lowest concentration of the compound that totally inhibited microbial growth (21).

To determine the effect of TF3 on test strains, a culture of each strain was treated with or without TF3 at sub-MICs and incubated for 36 h (22). The OD_600_ of the culture was monitored every 6 h by SpectraMax M2 Multifunction microplate reader (Meigu Molecular Instrument Co., Ltd., Shanghai, China).

### Extraction and detection of AHLs in WT and Δ*expR*

WT and ∆*expR* cultures treated with different concentrations of TF3 (7.8 mM, 15.6 mM, and 31.25 mM) were incubated for 24 h, and AHLs were then extracted according to Li et al. (18). After that, the extracted AHLs were added to each well (20 µL), which were punched in fresh agar plates containing CV026, and the plates were incubated at 30℃ for 24 h. The presence of AHL was detected by the appearance of the purple zone, and the level of AHL was quantified in terms of the size of the purple zone (23).

### The determination of motility assay of WT, Δ*lasI*, and Δ*expR*

The assay for *H. alvei* H4 motility (swimming and swarming) was performed as previously described (24) with slight modifications. In brief, WT, Δ*lasI*, and Δ*expR* were each incubated with 7.8 mM, 15.6 mM, and 31.25 mM TF3 overnight, and 3 mL of the culture was placed at the center of a swimming agar plate (agar 0.3%, tryptone 1%, and NaCl 0.5%) or swarming agar plate (Trypticase soy broth [TSB] medium, 0.5% agar). The plate was incubated for 48 h, and flagellar motility was determined by measuring the diameter (centimeter) of free cells (25). The twitching motility assay was performed exactly as described by Déziel et al. (26).

### Biofilm formation analysis

#### Biofilm biomass

Three strains were first tested for their ability to form biofilms within 72 h (27). Briefly, bacterial cultures and LB medium were transferred into 96-well polystyrene microplates at a ratio of 1:100, and biofilms were formed over 12, 24, 36, 48, 60, and 72 h. The formed mature biofilms were washed three times with sterile 1× phosphate-buffered saline (PBS), then fixed with 200 µL of methanol for 15 min, dried at 60℃, and stained with 200 µL of 0.1% crystal violet (CV) for 15 min. The plates were again washed three times with deionized water to remove excess dye and then dried at 60℃. The biofilm formed on the plates was dissolved by adding 200 µL of 33% acetic acid (per well) and then incubated for 20 min at room temperature. The absorbance of the plates was then measured at 590 nm using a spectrophotometer (Molecular Devices, San Francisco, CA, USA).

#### The viability of sessile cells in biofilms

The viability of the biofilm cells was determined using 3-(4,5-dimethylthiazol-2-yl)−2,5-diphenyl tetrazolium bromide (MTT) assay developed by Krom et al. (28). One milliliter of culture medium and 0.1 mL of 5 mg/mL MTT solution were added to each well and incubated at 30℃ for 4 h. After discarding the culture supernatant, 1 mL of DMSO was added to each well to dissolve MTT for 30 min. The optical density of each well was measured at 570 nm.

#### Enumeration of planktonic and sessile cells

CFU counts were used to determine the number of cultivable cells in planktonic culture and disrupted biofilms. Briefly, a total of 2 mL of three target bacterial culture biofilms with or without TF3 was prepared. After 24 h, the supernatant of each well was transferred to a centrifuge tube and serially diluted to obtain a planktonic cell suspension. The dilution ratios of 10^−5^, 10^−6^, and 10^−7^ of target strains were measured using LB agar plates. Furthermore, the number of sessile bacteria in biofilms was quantified using “bead vortexing method” (29). Adherent biofilms on glass tubes were washed with saline before adding 2 mL 0.9% saline solution and glass beads and vortexed vigorously to detach the biofilm cells. After serial dilutions of the isolated cells on LB agar plates, colonies of the target strains 10^−3^, 10^−5^, and 10^−7^ were counted in three dilutions.

### Real-time quantitative polymerase chain reaction assay

Real-time quantitative polymerase chain reaction (RT-qPCR) was carried out to determine the effect of TF3 on the expression of some QS-mediated genes (*cheA*, *fliC*, and *motA*). WT, Δ*lasI*, and Δ*expR* were each incubated with 7.8 mM, 15.6 mM, and 31.25 mM TF3, respectively, overnight until mid-log phase; then, the cells were harvested at the same OD_600 nm_ (around 0.7), and total RNA was extracted using a Tiangen RNA extraction kit (Tiangen Biotech, Beijing, China). The extracted RNA was converted to cDNA using a PrimeScriptIM RT Reagent Kit with gDNA Eraser (TaKaRa, Dalian, China). RT-qPCR was performed with a BIO-RAD MyiQTM2 Real-Time Detection System and SYBR Green PCR Master Mix (TaKaRa, Dalian, China) using the primers listed in Table 1. The 16S rRNA was used as an internal control, and the fold change of each target gene was determined using the 2^−∆∆Ct^ method as previously described (30).

**TABLE 1:** *H. alvei* H4 gene-specific primers used in RT-qPCR

Gene	Primer sequence(5′→3′）	Primer length
*16S rRNA*	F TAGCGGTGAAATGCGTAG	18 bp
	R TCGTTTACAGCGTGGACTA	19 bp
*cheA*	F ACGACGAAGTGGGATTGC	18 bp
	R CACGAGGCTGAAGGGATT	18 bp
*fliC*	F AGTTGGGACTCAAGCACAG	19 bp
	R TAGGGATGTTGCTCAGGTC	19 bp
*motA*	F TTCATGTTGCCGCTTACC	18 bp
	R ACCCGCAAGAAAGTGAAC	18 bp

### Molecular docking

The mechanism by which the test compound inhibited LasI*_Ha_*/ExpR was analyzed by Schrödinger Maestro 11.4. Two-dimensional maps were used to obtain the details of the interaction between the protein active site and the test compound. The molecular docking result was then visualized and analyzed by PyMoL software (1.3r1, DeLano Scientific LLC, South San Francisco, CA, USA).

### Molecular dynamics simulation

Molecular dynamics (MD) studies of protein-ligand complexes were performed following the method of Musyoka et al. (31). MD simulations were performed using the Gromacs 2018.4 program at a constant temperature and pressure. For the proteins, the mber14SB all-atom force field was applied, whereas, for all test compounds, the AMBER-based GAFF force field with the TIP3P water model was used. During the MD simulation, all bonds involving hydrogen atoms were constrained using the LINCS algorithm with an integration step size of 2 fs. Electrostatic interactions were calculated by the (Particle-Mesh Ewald) PME method. The truncation value for non-bonded interactions was set at 10 Å and updated every 10 steps. The V-rescale temperature coupling method was used to control the simulated temperature at 298.15 K, and the pressure was controlled at 1 bar by the Parrinella-Rahman method (32). First, the energy of the two systems was minimized by the steepest descent method to eliminate the contact between atoms that were too close. Then, the canonical ensemble (NVT) and constant-pressure, constant-temperature (NPT) equilibrium simulations were carried out at 298.15 K for 1 ns, respectively. Finally, the system was simulated by MD for 50 ns, and the conformation was saved every 10 ps. The visualization of the simulation results was done through the Gromacs inline program and Visual Molecular Dynamics program.

### Statistical analysis

Analysis of variance (ANOVA) was performed using SPSS Statistics 20.0, and differences between variables were tested by one-way ANOVA and Tukey’s test. All graphical data were drawn with Origin Pro 9.0. All experiments were replicated in triplicate, and all analyses were performed with three replicates.

## RESULTS AND DISCUSSION

### Construction of the 3D structure of LasI*_Ha_*

**Fig 1:**
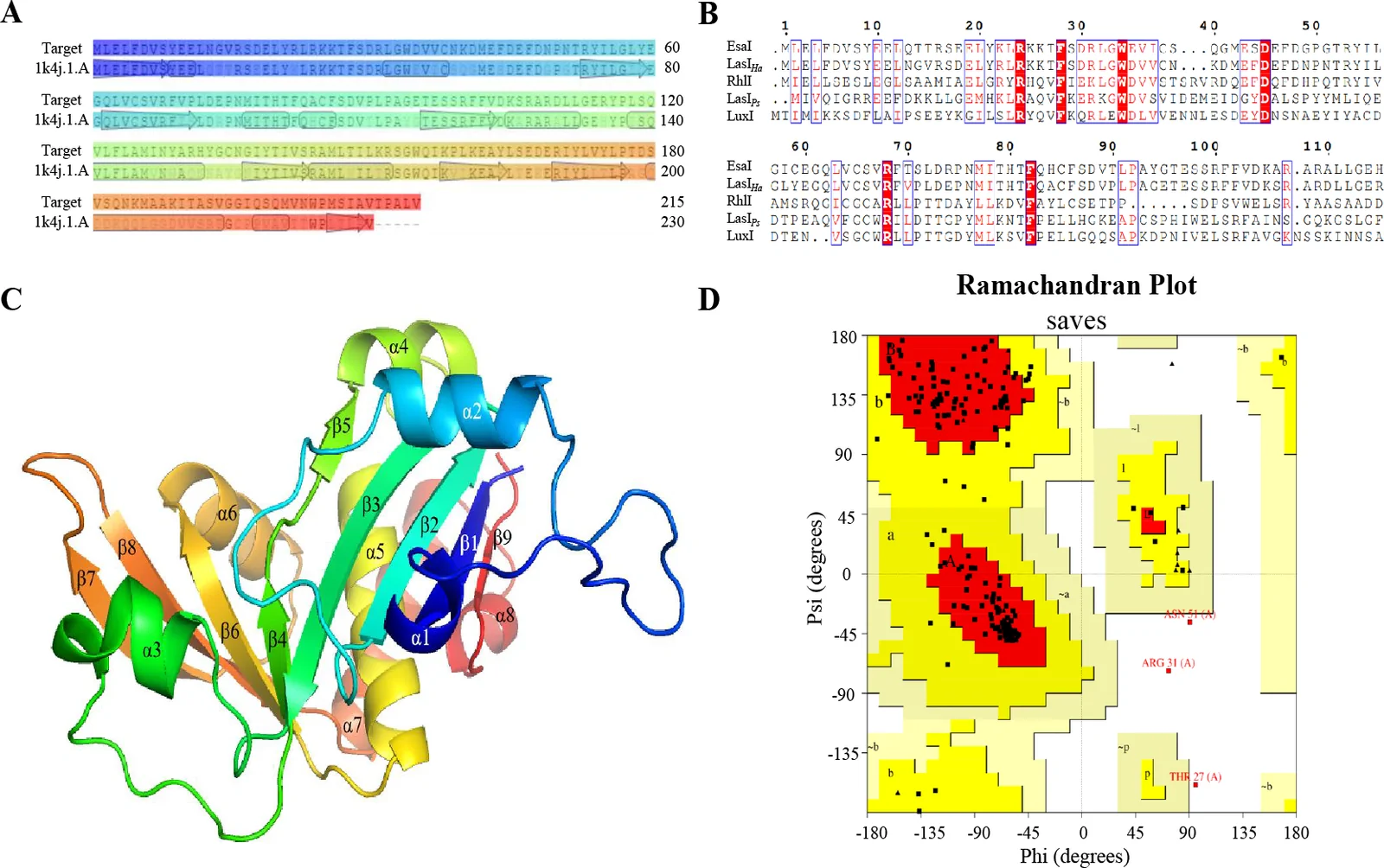
(**A**) Alignment of the template (1k4j.1.A) and target (LasI*_Ha_*) sequences. The color from blue to red indicates the N- to C-terminal positions of residues within the sequence. (**B**) Sequence alignment of the AHL-synthase EsaI, LasI*_Ha_*, RhlI, LasI*_Ps_*, and LuxI (protein sequence access address: https://www.uniprot.org/). Conserved residues are shaded, while residues in boxes are conservative substitution. (**C**) Ribbon representation of the 3D structure of LasI*_Ha_* obtained via modeling. Each colored portion of the structure corresponds to its location within the primary structure shown in **A**. (**D**) Ramachandran plot analysis of LasI*_Ha_* protein.

The 3D structure of LasI*_Ha_* was eventually obtained by homology modeling. First, known proteins with structures homologous to *H. alvei* H4 LasI*_Ha_* sequence were found by Blast, and those with highest homology were selected for full sequence comparison. Finally, proteins with more than 30% similarity to LasI*_Ha_* were used as templates to build the model, since a model built from such a criterion would well represent the actual structure of the target protein. Table 2 lists the templates with high GMQE and QMEAN scores as well as sequence identity (%). The template 1k4j.1.A showed the highest GMQE (0.83) and QMEAN (−1.83) scores and shared a high sequence identity (64.6%) with LasI*_Ha_*. Therefore, chain A of 1k4j was selected as the final template for the homology modeling of LasI*_Ha_*, and the sequence alignment of LasI*_Ha_* with the 1k4j template is shown in Fig. 1A.

**TABLE 2:** Three-dimensional structures and descriptions of *H. alvei* H4 LasI

Proteins	Template	Description	Seq similarity	Seq identity	GMQE	QMEAN	Model
LasI	1k4j.1.A	Acyl-homoserinelactone synthase EsaI	0.49	65.24%	0.81	−1.83	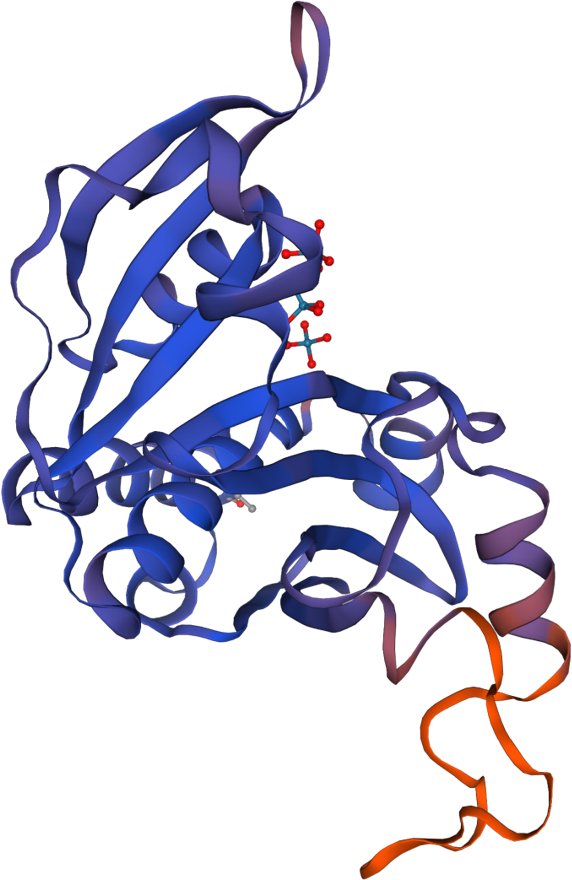
	5w8c.1.A	Autoinducer synthase	0.35	30.50%	0.41	−3.19	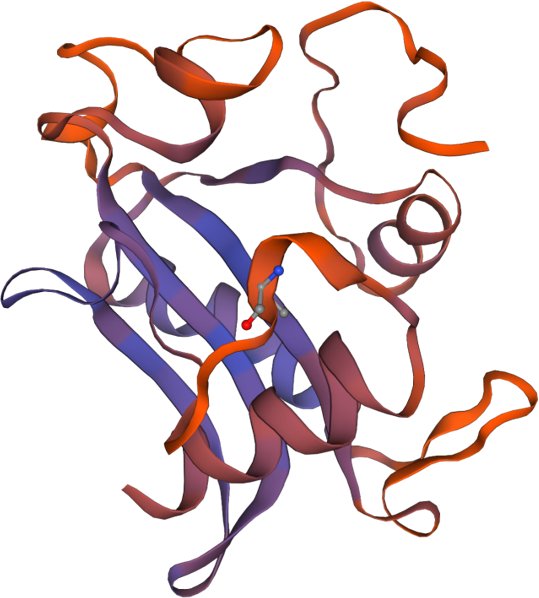
	5w8a.1.A	Autoinducer synthase	0.35	30.50%	0.41	−3.36	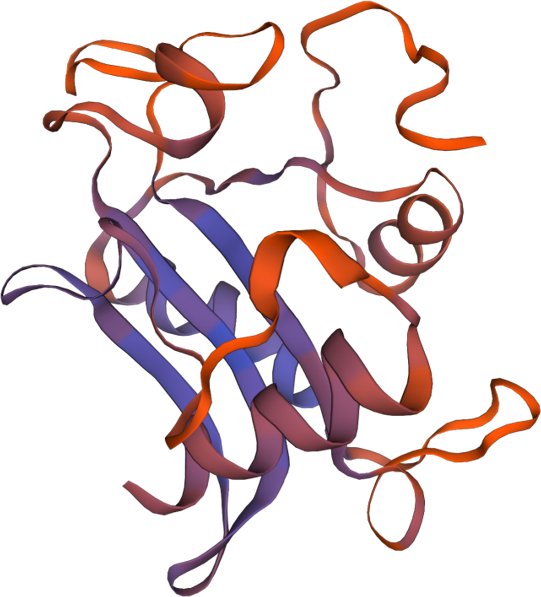

In addition, the alignment of *H. alvei* H4 LasI*_Ha_* with different AHL synthases revealed that Arg^23^, Phe^27^, Trp^33^, Asp^45^, Arg^68^, and Phe^82^ are highly conserved residues (Fig. 1B). According to the 3D structure of *P. aeruginosa* LasI*_Ps_* (12), Arg^23^, Phe^27^, and Trp^33^ form a closed S-adenosine-L-methionine substrate binding pocket. Compared with *P. aeruginosa* LasI*_Ps_*, the N-terminal region visible in the crystal structure of *P. stewartii* EsaI (13) has a more open conformation that makes up a highly mobile active site groove and is formed by the same three conserved residues, Arg^24^, Phe^28^, and Trp^34^ in this case. The remaining conserved residues, Asp^45^ and Arg^68^, aggregate to form an ion-pair network to stabilize the interactions within the N-terminal structural domain.

The predicted structure of *H. alvei* H4 LasI*_Ha_* also consisted of nine β chains surrounding eight α sheets in a highly distorted open conformation (Fig. 1C), and the function dictated by this structure could be predicted from the reported crystal structure of *P. aeruginosa* LasI*_Ps_*, which consists of three α helixes supporting a six-chain β sheet platform to form a V-shaped substrate-binding site. This results in a tunnel that can accommodate the acyl-ACP chain without any apparent conformational change, which is in contrast to the restricted hydrophobic pocket in EsaI. In short, the hydrophobic channel of LasI*_Ps_* might also provide valuable information for the design and/or detection of specific LasI-type inhibitors.

The Ramachandran plot analysis of LasI*_Ha_* showed a total of 98.4% of residues in the favorable and additional allowed regions, and 1.6% were in disallowed regions (Fig. 1D), indicating a good quality model for the predicted LasI*_Ha_* structure.

### Virtual screening results and compound selection for further experimental validation

**Fig 2:**
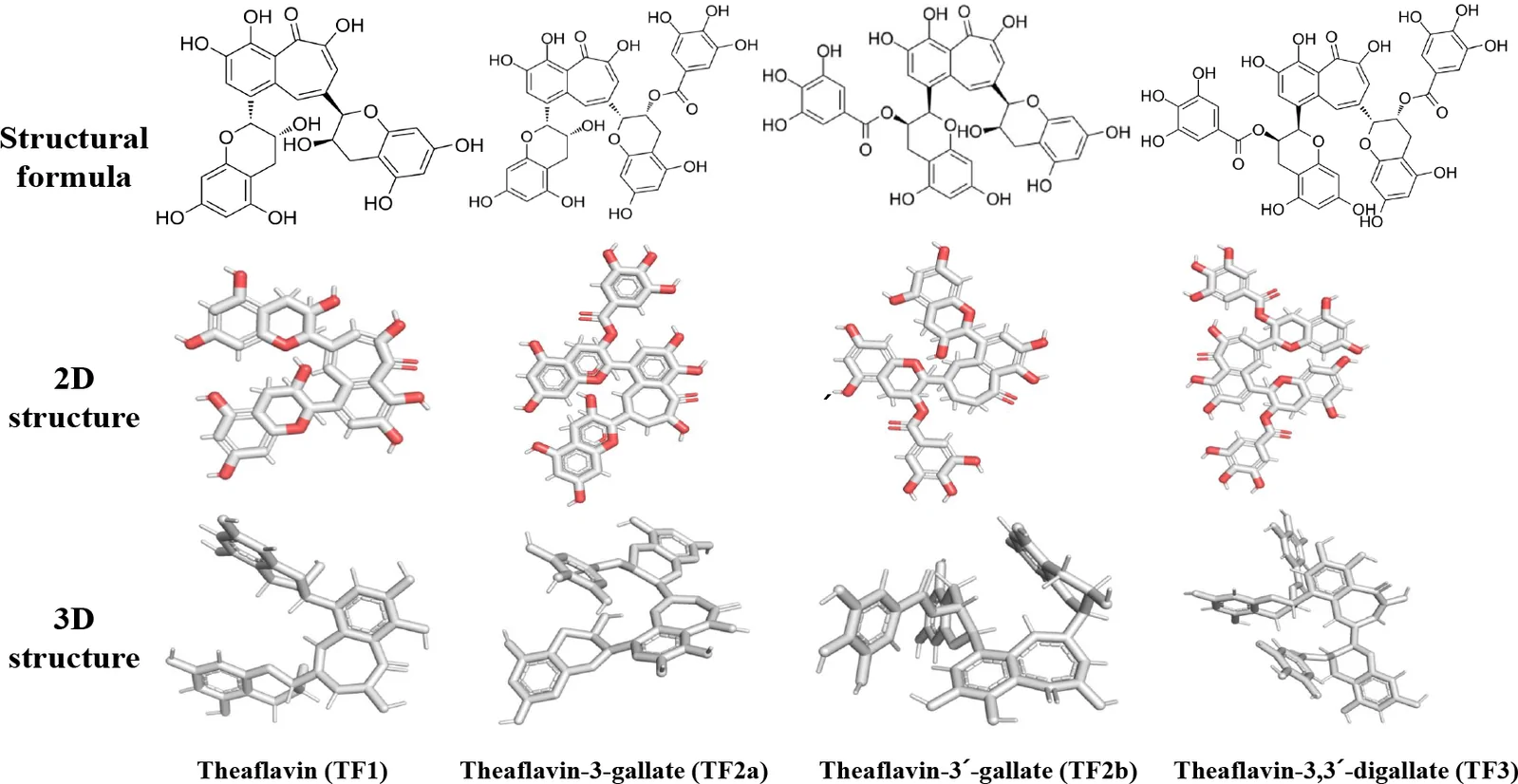
Structural formula and 2D and 3D molecular structures of theaflavin (TF1), theaflavin-3-gallate (TF2a), theaflavin-3´-gallate (TF2b), and theaflavin-3,3´-digallate (TF3).

The structure and scoring values of some of the top 200 compounds identified by virtual screening are shown in Table 3. The higher the absolute value of the molecular docking score, the stronger the binding force between the compound and protein. Among the top 200 compounds were two theaflavins, theaflavin-3,3´-digallate (TF3, ranked second) and theaflavin-3´-gallate (TF2b, ranked 53^rd^), which exhibited high docking scores as determined from the binding with LasI*_Ha_*. Notably, theaflavin has four variants (theaflavin [TF1], theaflavin-3-gallate [TF2a], TF2b, and TF3), but only TF3 and TF2b were listed in the top 200, and this may be due to incomplete coverage of the small molecule database. Thus, all four theaflavins were examined for their antibacterial and anti-QS activities, since we wanted to determine whether the conserved structures or some specific groups of these theaflavins might be important for their activities. The respective structural formula and 2D and 3D structures of four TFs are shown in Fig. 2. In addition, another 10 small molecules with high docking scores or safe for food application were also selected from the top 200 for further experimental verification (Table 3).

**TABLE 3:** QSIs identified from MCE bioactive compound library by the virtual screening with LasI as the target protein

Chemicals_Name	docking_score	CAS no.	Formula	Mw	Solubility
Poliumoside	−10.883	94079–81-9	C_35_H_46_O_19_	770.73	DMSO
Theaflavin 3,3'-digallate	−10.742	30462–35-2	C_43_H_32_O_20_	868.70	DMSO
Glutathione oxidized	−10.391	27025–41-8	C_20_H_32_N_6_O_12_S_2_	612.63	H_2_O
NADPH (tetrasodium salt）	−10.143	2646–71-1	C_21_H_26_N_7_Na_4_O_17_P_3_	745.42	H_2_O
Stevioside	−9.636	57817–89-7	C_38_H_60_O_18_	804.87	DMSO
Rubusoside9	−9.054	64849–39-4	C_32_H_50_O_13_	642.73	DMSO
NAD+	−9.028	53–84-9	C_21_H_27_N_7_O_14_P_2_	664.43	H_2_O
Eriocitrin	−8.953	13463–28-0	C_27_H_32_O_15_	596.53	DMSO
Disodium 5'-inosinate	−7.147	4691–65-0	C_10_H_11_N_4_Na_2_O_8_P	348.21	DMSO
Folic acid	−6.013	59–30-3	C_19_H_19_N_7_O_6_	441.40	DMSO
Sunset Yellow FCF	−5.748	2783–94-0	C_16_H_10_N_2_Na_2_O_7_S_2_	408.41	H_2_O

### MIC and anti-QS activity of four potential QSIs

The MIC of each test compound was measured to ensure the final concentrations of these compounds used in the experiments did not affect bacterial growth. All four TFs were found to display good antibacterial activity, with TF3 yielding the strongest activity and naturally, the lowest MIC value (125 µM) compared with TF1 (300 µM), TF2a (150 µM), and TF2b (600 µM). The other 10 compounds did not show any obvious antibacterial activity at the highest concentrations tested, 600 mM for the three *H*. *alvei* H4 strains and 2 mM for CV026 (Table 4).

**TABLE 4:** Minimum inhibitory concentration of the compounds that are potential QSIs

Target microorganism		*H. alvei* H4 WT	*H. alvei* H4 ∆*lasI*	*H. alvei* H4 ∆*expR*	CV026
MIC (mM)	Poliumoside	>0.6	>0.6	>0.6	>2
	TF3	0.125	0.15	0.15	>2
	TF1	0.3	0.6	0.6	>2
	TF2a	0.15	0.15	0.3	>2
	TF2b	0.6	0.3	0.6	>2
	Glutathione oxidized	>0.6	>0.6	>0.6	>2
	NADPH	>0.6	>0.6	>0.6	>2
	Stevioside	>0.6	>0.6	>0.6	>2
	Rubusoside	>0.6	>0.6	>0.6	>2
	NAD+	>0.6	>0.6	>0.6	>2
	Eriocitrin	>0.6	>0.6	>0.6	>2
	Disodium 5'-inosinate	>0.6	>0.6	>0.6	>2
	Folic acid	>0.6	>0.6	>0.6	>2
	Sunset Yellow	>0.6	>0.6	>0.6	>2

**Fig 3:**
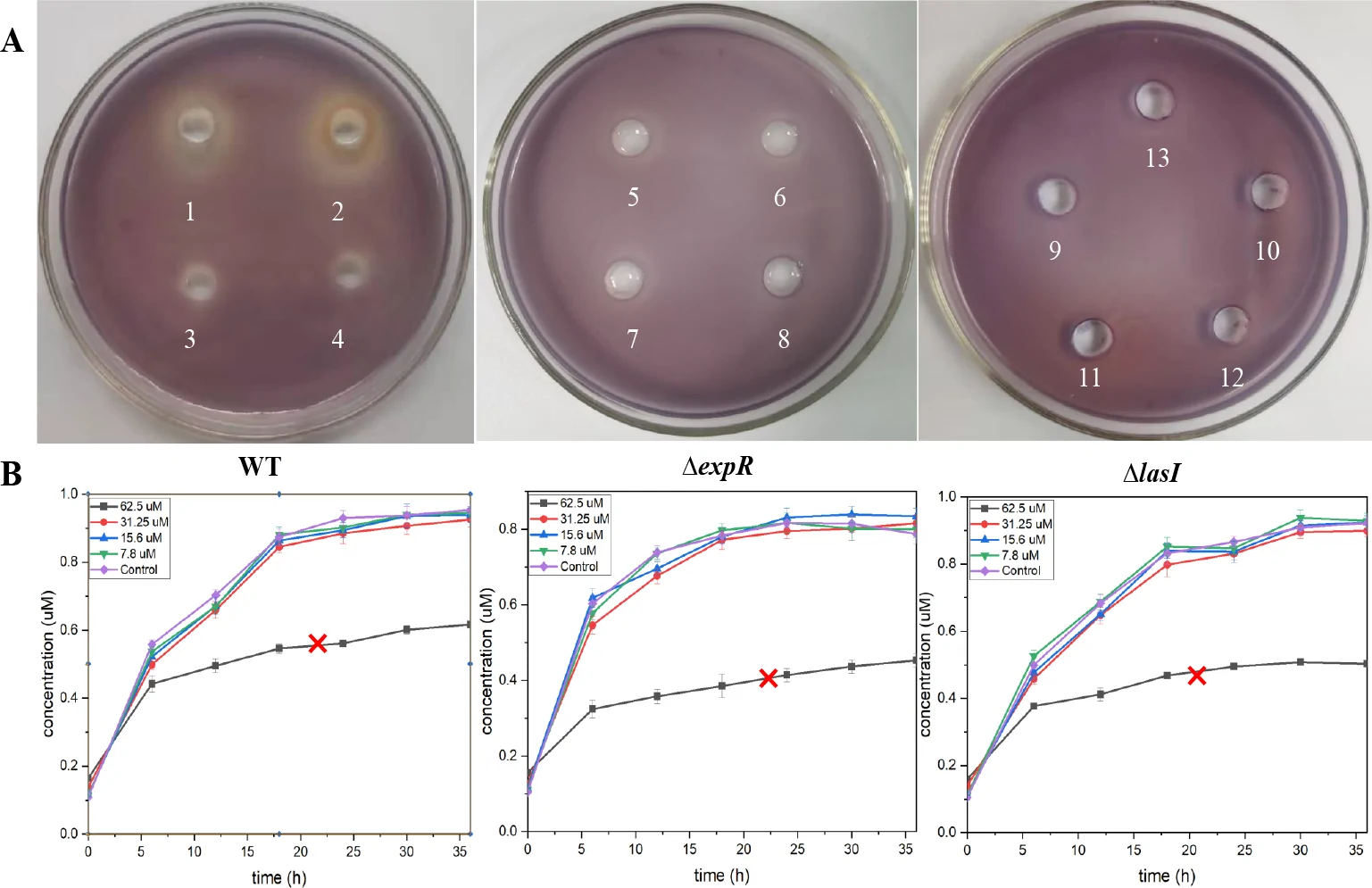
(**A**) Effects of the potential QSI compounds identified by the virtual screening on the inhibition of violacein production in *Chromobacterium violaceum* (CV026) (1), poliumoside (2), theaflavin-3,3’-digallate (3), glutathione oxidized (4), NADPH (5), stevioside (6), rubusoside (7), NAD+ (8), eriocitrin (9), disodium 5’-inosinate (10), folic acid (11), Sunset yellow (12), 30% DMSO (13), and sterile water. (**B**) Effects of TF-3 on the growth of *H. alvei* H4 WT, ∆*lasI*, and ∆*expR* at different concentrations. All data were expressed as means ± SD (*n* = 3). The red X means that when the concentration of TF3 was 62.5 µM, it has started to affect the growth of three strains.

The above experiments showed promising MIC values for the four TFs, while MIC values did not correlate with whether the small molecule compounds themselves had anti-QS activity, so further evaluation of the anti-QS activity of all tested compounds is needed. CV026 is unable to produce AHLs but can bind to exogenous short-chain AHLs, resulting in the secretion of violacein. From the plate assay, a relatively obvious distinct cloudy halo was observed around the wells containing poliumoside or TF3 (Fig. 3A), whereas only faint halos appeared around the wells containing stevioside, rubusoside, or NAD^+^. Since the size of the halo is a direct indication of the level of AHL in the sample, the degree of translucence and the size of the halo would reflect the anti-QS activity of the test compound (33). The results suggested that TF3 exerted excellent anti-QS activity, and since the other compounds did not affect the level of violacein in the plate because they lacked the anti-CV026 activity, these compounds were excluded from the subsequent QS phenotype experiments. TF3, a type of theaflavins derived from black tea (34), is widely considered to be the most effective bioactive component of TFs, as it is capable of acting as an antioxidant, antibacterial, and antitumor agent (35).

**Fig 4:**
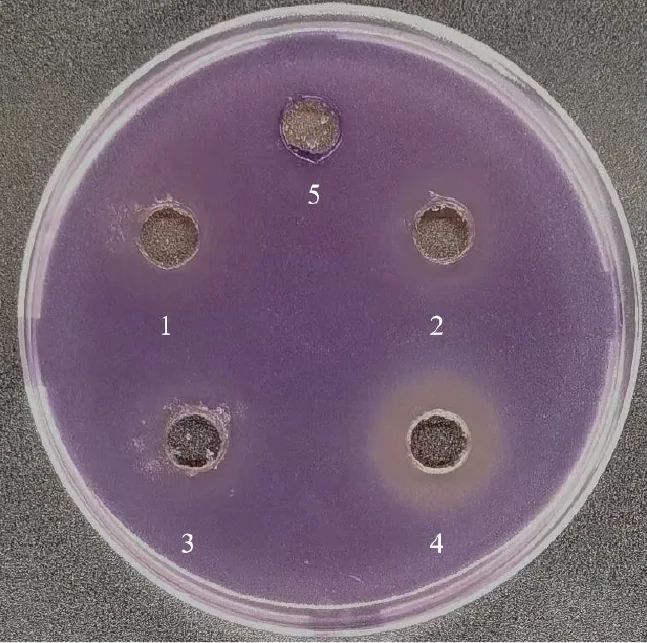
Inhibitory effects of the four TFs on violacein production in *Chromobacterium violaceum* (CV026) (1), theaflavin (2), theaflavin-3-digallate (3), theaflavin-3’-digallate (4), theaflavin-3,3’-digallate (5), and 30% DMSO.

Again, TF3 was found to display a stronger inhibitory effect on violacein production by CV026 than the other three TFs (Fig. 4), indicating its stronger anti-QS effect. Interestingly, the structures of the four TFs differ only in the position and number of gallic acid at the C-3- and C-3´ positions, but the overall 3D structures of these molecules are very different (Fig. 2). Since the structure of a molecule also determines its function, the different antibacterial and anti-QS activities observed for these four TFs could be attributed to the differences in their 3D structures. Both TFs and polyphenols (TP) are polyphenols that are found in tea, especially black tea, and TPs have been considered a novel class of non-antibiotic QSIs (36). However, the exact structure-function relationship of TFs responsible for their anti-QS active effects had not been previously determined, so it was important to further analyze whether the functional differences of the four TFs were due to the polyhydroxyl group of the theaflavin or gallic acid component.

### Effect of TF3 on AHL synthesis

**Fig 5:**
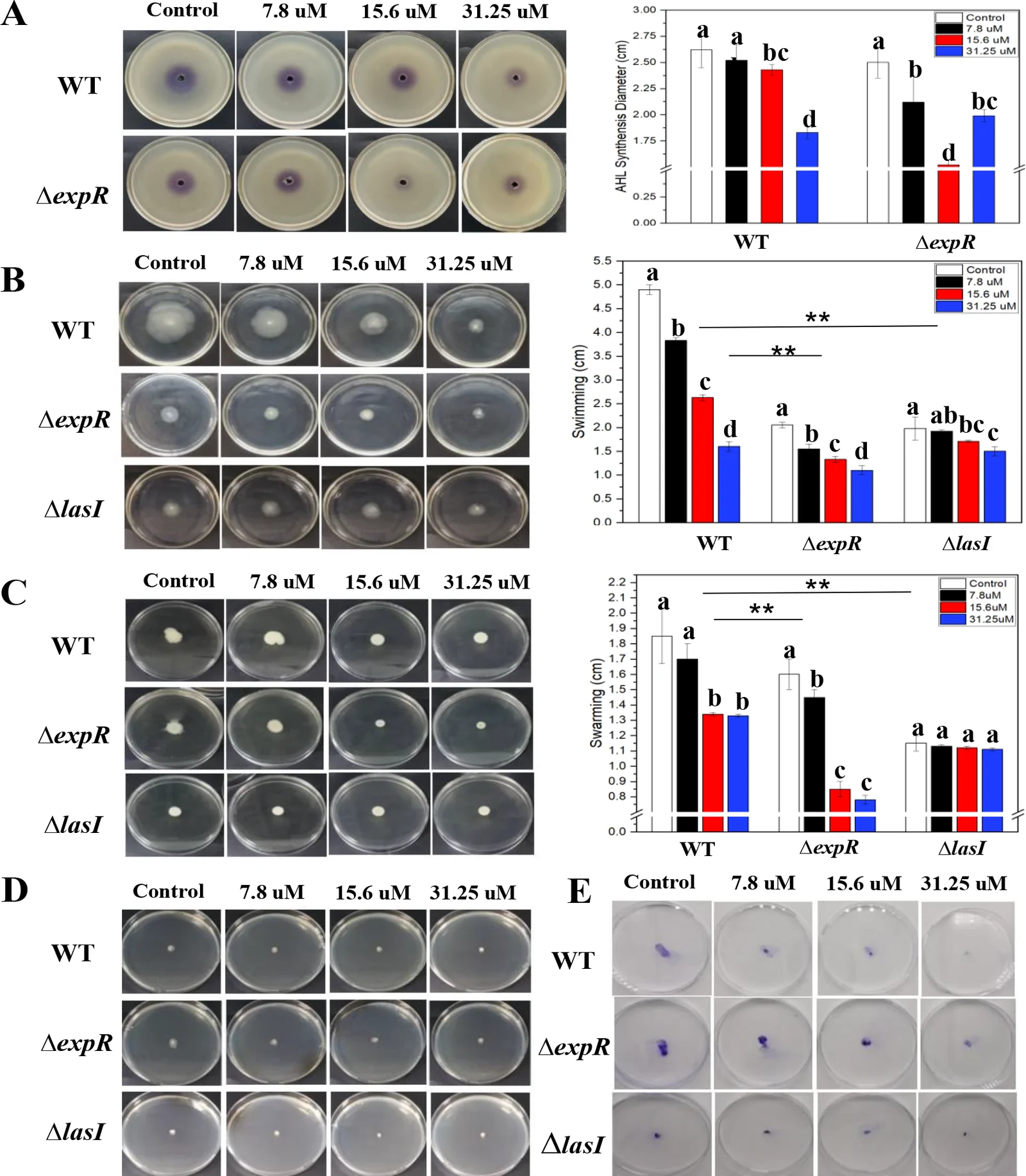
Effect of different concentrations of TF3 on the anti-QS phenotype of *H. alvei* H4. (**A**) Effect of TF3 on the production of AHLs in WT and Δ*expR*. The plot compares the synthesis of AHLs in terms of the zone diameter. Inhibition of the swimming motility (**B**) and swarming motility (**C**) of WT, Δ*expR*, and Δ*lasI*. In both panels **B** and **C**, the plots compare the extent of motility in terms of the migration distance of the bacteria from the point of inoculation. (**D**) Inhibition of twitching motility of WT, Δ*expR and* Δ*lasI*. (**E**) Plate surface map of twitch area stained with crystal violet after the removal of agar from the plate in panel **D**. Statistical significance (**) was considered at *P* < 0.01 level, while a, b, c, and d represent significant differences between groups.

To verify the inhibitory effect of TF3 against *H. alvei* H4 was primarily a result of it targeting LasI*_Ha_*, the effect of TF3 on AHL production in ∆*expR* was compared with its effect on the WT. Analysis of the growth of *H. alvei* H4 in the presence of TF3 revealed that inhibition of growth of either WT or the two mutants did not occur except at 62.5 µM concentration of TF3. (Fig. 3B). Since ∆*lasI* could not produce AHL (data not shown), it was not subjected to an AHL assay analysis. In the AHL assay, CV026 could respond only in the presence of exogenous AHLs and, after which, produced the characteristic violet pigment, violacein. Thus, its absence is a direct indication of the absence of AHL. In WT, the production of violacein was reduced, and the higher the TF3 concentration, the stronger the reduction, as revealed by a weaker purple zone in the plate (Fig. 5A). As for ∆*expR*, the reduction of violacein production was most obvious at 15.6 mM TF3, since more violacein was produced when the concentration of TF3 was increased to 32.25 mM, and this was rather unexpected. Geng et al. (37) found that treatment of *P. aeruginosa* with different sub-MIC concentrations of luteolin reduced the production of OdDHL, but surprisingly, the effect of 100 µM luteolin was much greater than the effect of 200 µM. The authors considered the higher concentration of luteolin may affect the activity of RsaL (a transcriptional repressor of the *lasI* gene), which can then further affect the level of OdDHL, since RsaL can also regulate the production of OdDHL (38). We have previously identified several DNA-binding transcriptional repressors (AcrR, fur, DeoR, and PurR) in *H. alvei* H4 (18). We speculated that knockout of the *expR* gene allowed the TF3-LasI*_Ha_* complex to exert an inhibitory effect on AHL production at 7.8 mM and 15.6 mM, but when the TF concentration increased to 31.25 mM, it may have reached the threshold concentration that led to the relief of the repressive effects of those transcriptional repressors on the *lasI* gene, thereby prompting an increase in the expression of LasI*_Ha_* and, hence, more production of AHL. The verification of this speculation requires further study.

### Effect of TF3 on the motility of *H. alvei* H4

Flagellar motility such as swimming and swarming is the main form of locomotion for most mobile bacteria, with the benefit of helping bacterial cells obtain nutrients, form biofilms, and colonize a surface (39). Similarly, twitching motility based on type IV pilus has also been shown to be required for the initial attachment of bacterial cells during biofilm formation (40). As for *H. alvei* H4, its motility was clearly inhibited by TF3, but the effect of TF3 appeared to vary for the different types of motilities and for the different bacterial strains. All three strains exhibited progressively reduced swimming motility with increasing TF3 concentrations, but the wild type was most affected (Fig. 5B). Only the WT and ∆*expR* showed severe loss of swarming motility with increasing TF3 concentrations (Fig. 5C). Furthermore, TF3 exerted a much stronger inhibition on the swarming motility of ∆*expR* than on the swarming motility of WT at 15.6 mM and 31.25 mM compared with 7.8 mM (Fig. 5C). The swarming motility of ∆*lasI* appeared to be unaffected by TF3 because it did not change significantly upon the addition of TF3. The reduction in swimming and swarming motility was reflected by a reduction of the migrated distance around the point of bacterial inoculation, and in this study, a concentration-dependent decrease in migration distance was observed for both swarming and swimming in WT and ∆*expR*. Since swarming is driven by multiple flagellar while swimming requires only one flagellum (41), we speculated that TF3 might not only affect the number or activity of the flagella of individual cells but could also affect the collective movement of a large number of cells. In addition, the inhibitory effect of TF3 on the motility of ∆*lasI* was relatively small, and there were no significant differences among the different concentrations of TF3. This may also indirectly verify that TF3 could exert a better inhibitory effect by binding to LasI*_Ha_*. Different motility phenotypes correspond to different activation mechanisms. Swimming motility is a mode of bacterial motility driven by rotating flagella, and it occurs when individual cells move in a liquid environment, whereas swarming motility is operationally defined as rapid multicellular bacterial surface motion driven by rotating flagella (41). Different from the inhibition of motility by TF3, some studies also observed motility inhibition by other phenolic compounds, like proanthocyanidins and tannins that completely inhibited swarming, but did not prevent swimming, and trans-resveratrol that was more active against swarming than against swimming motility (42), further suggesting that these movement phenotypes have different activation mechanisms and the inhibitory effects of different phenolics on motility were also not completely similar.

Furthermore, ∆*lasI* formed a smaller and denser zone at the plate-agar interface than the WT and ∆*expR* when the cells punctured the agar layer at the sample spots (Fig. 5D). In general, none of the three strains had strong twitching motility. Crystalline violet staining performed on the cells that remained on the plate after the agar had been scraped off indicated that the attached cells were closely associated with the twitchy region (Fig. 5E). Notably, the ability of each strain to attach to the plate surface was diminished after TF3 treatment, as shown by the smaller twitch zones in the plate before the agar was removed. The change in twitching motility exhibited by the bacterial cells was more clearly visualized after staining with crystal violet. TF3 could, therefore, affect type IV pilus or the related type II secretion system.

### Effect of TF3 on the biofilm formation of *H. alvei* H4

**Fig 6:**
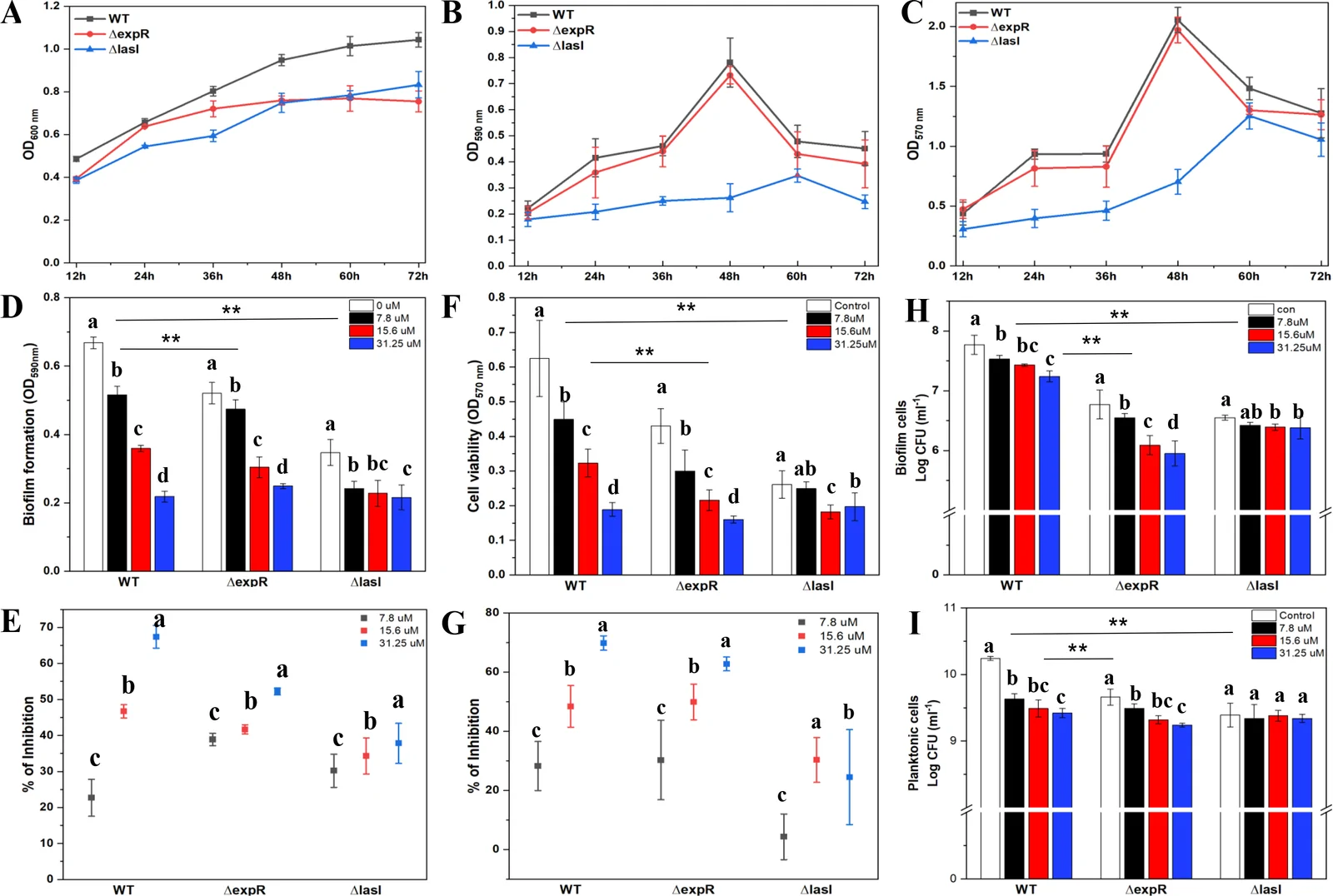
(**A, B, and C**) Dynamic formation of biofilms formation of *H. alvei* H4 WT, ∆*lasI*, and ∆*expR* without TF3. (**A**) The OD_600 nm_ value of bacterial growth of WT, ∆*lasI*, and ∆*expR*. (**B**) The OD_590 nm_ value of biofilm formation amount of WT, ∆*lasI*, and ∆*expR*.. (**C**) The OD_570 nm_ value of biofilm cell viability of WT, ∆*lasI*, and ∆*expR*. (**D**) Effect of TF3 on the biofilm formation by CV assay. (**E**) Percentage of inhibition of TF3 on biofilm formation in WT, Δ*expR*, and Δ*lasI*. (**F**) Effect of TF3 on the cell viability by MTT assay. (**G**) Percentage of inhibition of TF3 on cell viability in WT, Δ*expR*, and Δ*lasI*. (**H**) Effect of TF3 on the biofilm cells by enumeration assay. (**I**) Effect of TF3 on the planktonic cells by enumeration assay. Statistical significance (**) was considered at *P* < 0.01 level, while a, b, c, and d represent significant differences between groups.

Bacterial biofilm formation is a dynamic process with distinct phases of development (43). In this study, the CV assay was used to examine the dynamics of biofilm development of *H. alvei* H4 WT, Δ*expR*, and Δ*lasI* during 72-h incubation (Fig. 6A–C). The results showed that WT and Δ*lasI* continued to grow up to 72 h, whereas Δ*expR* flatten after 48 h (Fig. 6A). Both Δ*expR* and Δ*lasI* showed lower biofilm formation than WT, with a more pronounced decrease in Δ*lasI* in particular (Fig. 6B), consistent with previous laboratory result (6). Furthermore, previous studies have shown that cell viability was closely related to biofilm formation (44), and the differences in these strains in reaching the maturation stage were highly attributed to strain heterogeneity (45). The results showed that biofilms of WT and Δ*expR* grew rapidly with increasing incubation time and reached a maximum at 48 h (Fig. 6B) when the viability of their corresponding biofilm cells also reached a maximum (Fig. 6C), and both biofilm formation and biofilm activity of Δ*lasI* reached a maximum at 60 h. Our experimental results also confirmed the above view (Fig. 6B and C). We also observed that the biofilm of all three strains decreased after 60 h of incubation, indicating that the biofilm started to disintegrate. Throughout the biofilm formation process, we chose the biofilm incubated for 24 h to study the inhibitory effect of exogenous addition of TF3 on WT, Δ*expR*, and Δ*lasI*.

Independently of the fact that Δ*expR* and Δ*lasI* formed less biofilm than WT, it showed that addition of different concentrations of TF3 respectively inhibited biofilm formation in WT, Δ*expR*, and Δ*lasI*, and the reduction in biofilm amount occurred in a concentration-dependent manner (Fig. 6D), with the highest inhibition rate of 67.4% in WT by 31.25 µM TF3 (Fig. 6E). MTT assay was employed to explore the cell viability in WT, Δ*expR*, and Δ*lasI* biofilms. The biofilm cell viability of Δ*expR* and Δ*lasI* was significantly lower than that of WT, while the addition of sub-MICs of TF3 significantly reduced the OD_570 nm_ values of the three bacteria (Fig. 6F). The percentage of inhibition of cell viability by TF3 was high, with 31.25 µM of TF3 inhibiting WT and ∆*expR* by more than 60% (Fig. 6G), which was the same trend as the amount of biofilm formation (Fig. 6E). Furthermore, since biofilm is a dynamic formation process in which sessile and planktonic cells are inter-converted (43), we therefore further determined the effect of adding TF3 on sessile and planktonic cells in the solution. Analysis of this assay revealed that WT had more sessile and planktonic cells than Δ*lasI* and ∆*expR* (Fig. 6H and I). After the addition of sub-MICs of TF3, WT and ∆*expR* inhibited sessile and planktonic cells in the biofilm in a concentration-dependent manner, and Δ*lasI* appeared to be unaffected by TF3 because it did not change significantly upon the addition of TF3, which corresponds to the results in Fig. 6D and F and is also consistent with the trend in aforementioned motility experiments.

### Effect of TF3 on the expression of QS-regulated genes

**Fig 7:**
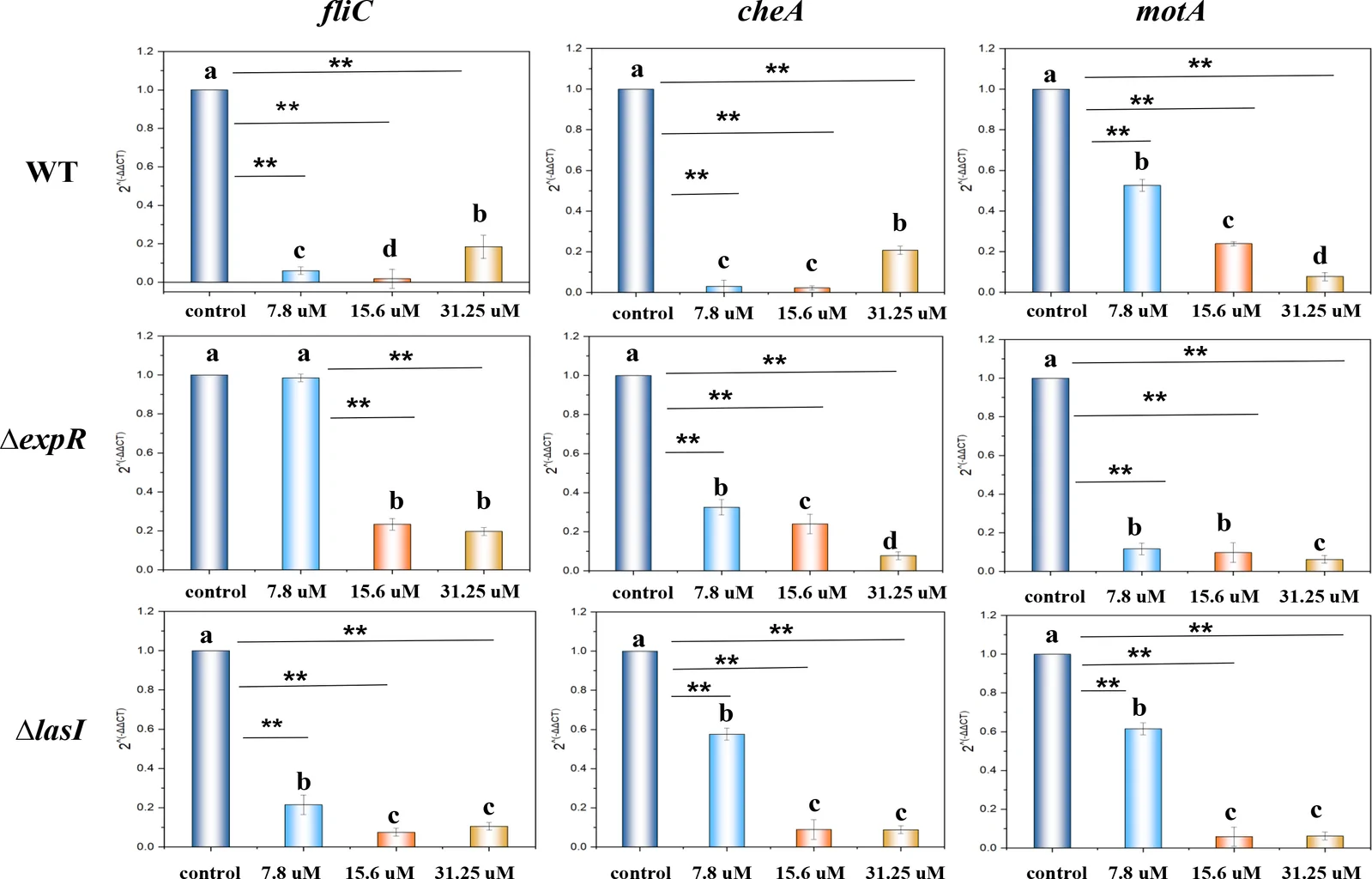
Effect of TF3 on the relative expression levels of some QS-related genes in *H. alvei* H4. WT and the two mutants were treated with different concentrations of TF3, and the mRNA levels of the QS-related genes (*fliC*, *cheA*, and *motA*) relative to the non-treated cells (control) were determined by RT-qPCR. Statistical significance (**) was considered at *P* < 0.01 level, while a, b, c, and d represent significant differences between groups.

RT-qPCR was used to determine the expression of QS-related genes in *H. alvei* H4 as a means to examine the effect of TF3 on QS at the transcriptional level. Three QS-related motility factors, *fliC*, *cheA*, and *motA*, were chosen for this analysis, and their primers were all optimized to 90–110% efficiency using the standard curve of DNA. Overall, the results revealed a significant reduction in *fliC*, *cheA*, and *motA* mRNA levels in both WT and the two mutants after exposure to increasing concentrations of TF3 (Fig. 7), indicating the strong inhibitory effect of TF3 on the expression of these genes, and this inhibitory effect was also reflected in the weakened motility of the bacterial cells (Fig. 5). Surprisingly, the inhibitory effect of TF3 on these genes was not entirely concentration dependent, at least in the case of the WT, since the reduction in *fliC* and *cheA* mRNAs was much less severe at the highest concentration (31.25 mM) of TF3. As for ∆*expR*, the effect of TF3 on the mRNA levels of *fliC*, *cheA*, and *motA* was quite similar between 15.6 mM and 31.25 mM, as both concentrations reduced the mRNA levels to a similar degree. The RT-qPCR data clearly showed the inhibitory effect of TF3 on the expression of the QS-mediated genes. Studies have shown that, in *Escherichia coli*, the *fliC* gene codes for a flagellar protein (FliC) required for the assembly of flagellar composition, whereas MotA, the product of the *motA* gene, in a component of the flagellar motor (41, 46), while CheA, the product of the *cheA* gene, is a histidine kinase involved in most of the signal transduction in bacteria. When a chemotactic receptor senses an environmental signal and triggers a stimulus in response, the stimulus signal is transmitted to the flagellar motor via CheA and CheY (47). Therefore, in a complex environment, flagellated bacteria will rotate clockwise and counterclockwise via the flagellar motor to achieve bacterial chemotaxis. We speculated that the proteins encoded by these genes in *H. alvei* H4 may also perform similar functions to the corresponding proteins in *E. coli*. The data suggested that after the inhibitory effect of the exogenous inhibitor reached a certain threshold, a higher concentration of inhibitor would have no further inhibitory effect on the expression of these genes. It also suggested that other factors may be at play, giving rise to a more complex regulatory network of motility, which requires further study to gain more insight.

### QS inhibitory mechanism analysis of TF3 binding LasI*_Ha_* by molecular docking

**Fig 8:**
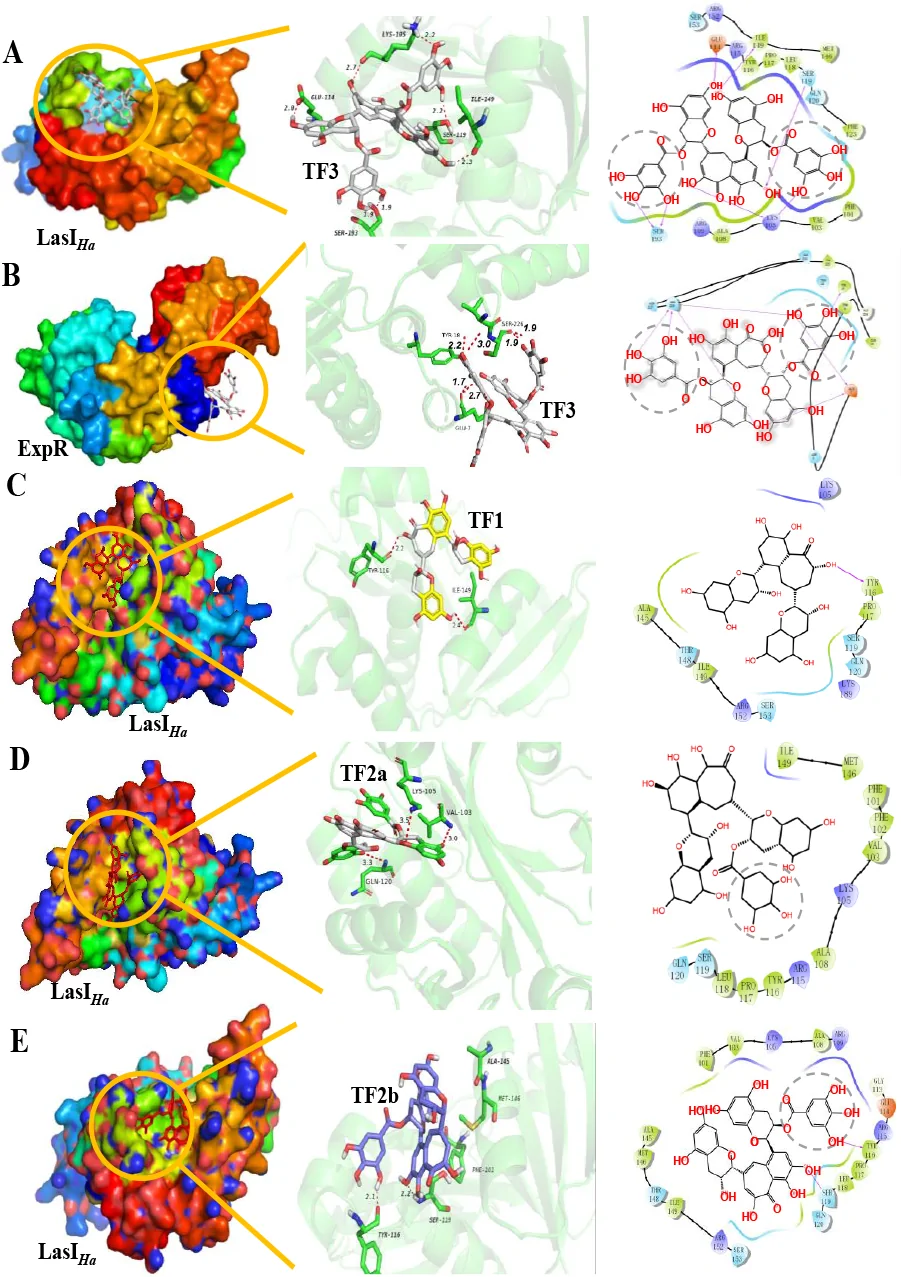
Molecular docking analysis of the binding of TF3 and other TFs with LasI*_Ha_* and ExpR. (**A**) TF3- LasI*_Ha_* complex. (**B**) TF3-ExpR complex. (**C**) TF1-LasI*_Ha_* complex. (**D**) TF2a-LasI*_Ha_* complex. (**E**) TF2b-LasI*_Ha_* complex. Each inhibitor-protein complex is shown as a surface representation mode, followed by a close-up view of the binding between the inhibitor and residues within the binding pocket of the protein in both ribbon and two-dimensional representations. The gallic acid structure is circled by the gray dashed circles in the two-dimensional diagram. All hydrogen bonds between TFs and respective residues in the protein are indicated in red dashed lines.

To determine the molecular basis of the interaction between TF3 and LasI*_Ha_*, molecular docking was used to probe the binding between the two molecules. Molecular docking techniques are widely used in the field of structural molecular biology and in the screening and development of new drugs (48) to identify the binding modes or static interaction forces between ligands and proteins. The result obtained from molecular docking revealed clear ligand-protein interaction for the TF3-LasI*_Ha_* complex. Fig. 8A shows the best model for the TF3-LasI*_Ha_* complex, with specific hydrogen bonds formed between TF3 and Glu^114^, Ile^149^, Ser^119^, Lys^105^, and Ser^193^, and that these bonds were contributed by the hydroxyl groups of both the theaflavin and gallic acid components of the TF3 structure. The ligand-protein interaction in the TF3-LasI*_Ha_* complex depicted by the two-dimensional interaction map was clearly illustrated. The hydrogen bond lengths formed between TF3 and LasI*_Ha_* ranged from 1.9 to 2.7 Å, with the TF3-Ser^193^ bond (1.9 Å) and TF3-Glu^114^ bond (2.0 Å) being stronger than the other hydrogen bonds. Since the shorter the bond length, the lower the resistance and the more stable the structure, the hydrogen bonds between TF3 and Ser^193^ and Glu^114^ were considered the most stable, and they may play an important role in the interaction between LasI*_Ha_* and TF3. In the TF3-LasI complex, TF-3 was wrapped in the hydrophobic pocket around the active pocket of LasI*_Ha_*, and this pocket consisted of 11 amino acids, including Phe^101^, Val^103^, Ala^108^, Arg^109^, Arg^115^, Tyr^116^, Pro^117^, Leu^118^, Met^146^, Arg^152^, and Ser-^153^. This might suggest the involvement of strong hydrophobic interaction in the protein-ligand complex, enabling the protein and ligand to bind more stably and further enhancing the affinity between protein and ligand. This result was supported by subsequent molecular dynamics data. Similarly, TF3 was also docked with ExpR, the AHL-binding regulator (Fig. 8B), and according to the 2D plot, TF3 formed long-range hydrogen bonds with only two amino acid residues of ExpR, Ser^226^ and Glu^7^, and such binding did not seem to result in a stable complex.

To further verify that the interaction between TF3 and LasI*_Ha_* was specific rather than a result of general binding between LasI*_Ha_* and a TF molecule, the binding between LasI*_Ha_* and other TFs was also carried out with molecular docking. The 3D binding patterns and the corresponding 2D plots of these TF-LasI*_Ha_* complexes are shown in Fig. 8C through E. One hydrogen bond was found between TF1 and LasI*_Ha_* (Tyr^116^), whereas no hydrogen bond between TF2a and LasI*_Ha_*, while two hydrogen bonds were found between TF2b and LasI*_Ha_* (Tyr^116^ and Ser^119^). With TF2b, in addition to the two hydrogen bonds, it also participated in hydrophobic interaction with a number of other residues, such as Ala^145^, Met^146^, Val^103^, and Phe^101^. Taken together, the result indicated a stronger and more specific interaction between LasI*_Ha_* and TF3 compared with those between LasI*_Ha_* and other TFs, based on the number of hydrogen bonds formed between the protein and ligand, as well as the binding conformation. Such a specific interaction may be related to the number and location of gallic acids in the structure of TF3, since it contains more hydroxyl groups that could more readily form hydrogen bonds with LasI*_Ha_* than the hydroxyl groups on the theaflavin component. Notably, gallic acid itself has no anti-QS activity, whereas propyl gallic acid, which is obtained by replacing the H on the -COOH group in gallic acid with a propyl group, does have QS inhibitory activity (49).

The stronger interaction between TF3 and LasI*_Ha_* demonstrated a more selective binding between TF3 and LasI*_Ha_*. Obviously, under a given concentration of TF3, LasI*_Ha_* would be more affected by TF3 compared with ExpR, allowing TF3 to interfere with the QS signaling by binding to LasI*_Ha_* and inhibiting its activity, consequently reducing the production of AHLs. This was consistent with the stronger inhibition of QS activity of WT and Δ*expR* compared with Δ*lasI*.

### QS inhibitory mechanism of TF3 as analyzed by molecular dynamics simulation

Finally, the overall conformational and stability changes of TF3 upon binding to LasI*_Ha_* and the effect of solvent on the stability of this system were analyzed via MD simulations. Specifically, a 50-ns molecular dynamics simulation of the LasI*_Ha_*-TF3 complex was performed under physiological conditions to understand the dynamic mechanism of the LasI*_Ha_*-TF3 interaction.

**Fig 9:**
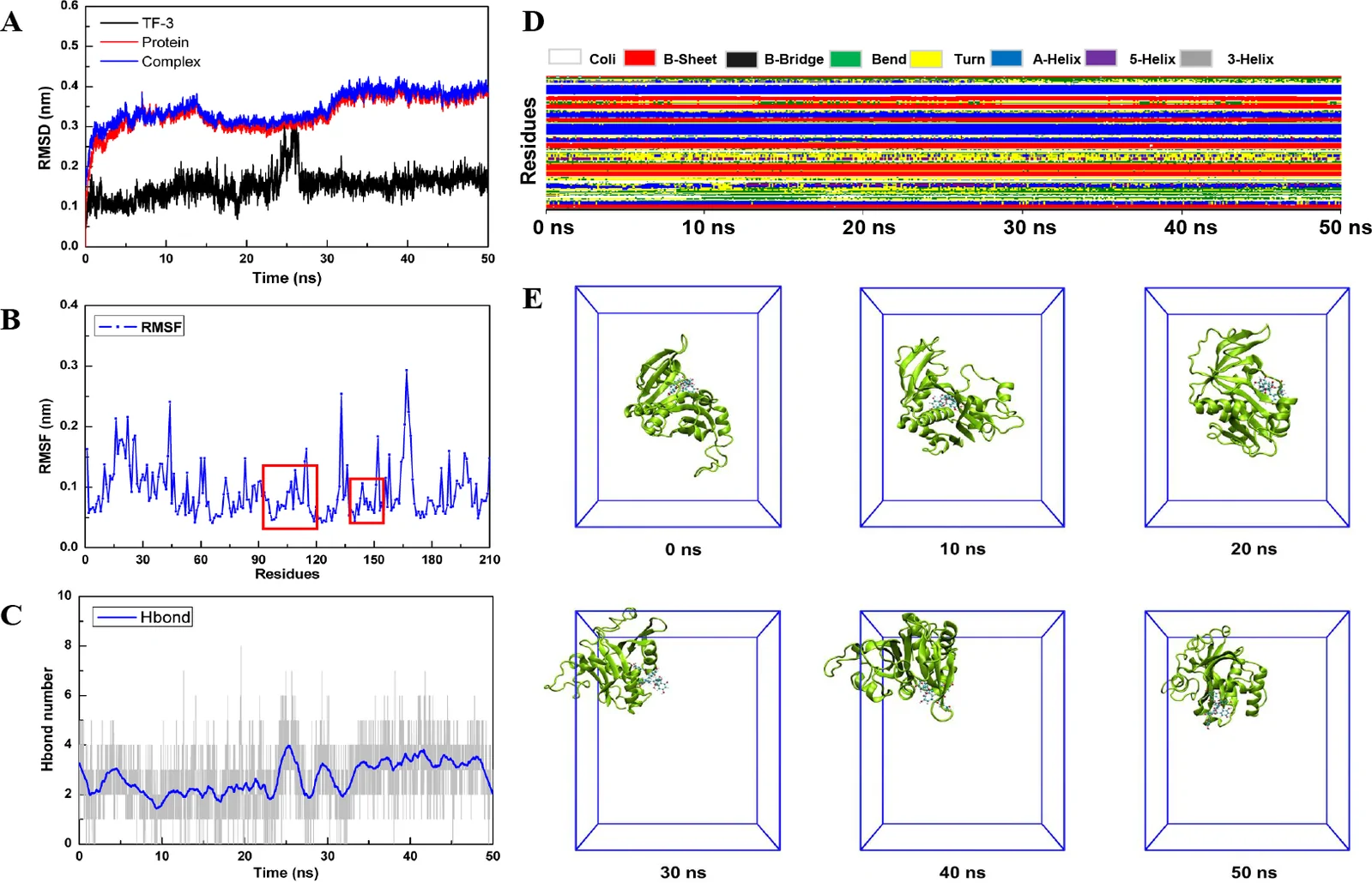
Molecular dynamics of LasI*_Ha_* upon TF3 binding. (**A**) RMSD of LasI*_Ha_* alone and in the presence of TF3 as a function of simulation time. (**B**) Root mean square fluctuation (RMSF) of LasI*_Ha_* upon TF3 binding. (**C**) Number of intermolecular hydrogen bonds between LasI*_Ha_* and TF3 as a function of simulation time. (**D**) Changes in LasI*_Ha_* secondary structure in the LasI*_Ha_*-TF3 complex as a function of simulation time. Time frames are shown on the X-axis, and amino acid residue numbers are shown on the Y-axis. Coil represents the loop or random coil; B-sheet and B-bridge represent the β fold; A-helix, 5-helix, and 3-helix all represent the α-helices; and turn and bend represent the β-turn. (**E**) Configuration of TF3 around the LasI*_Ha_* protein during 50 ns of MD simulation; the blue border is the box of the simulation system.

The RMSD value of the LasI*_Ha_*-TF3 system rose rapidly from the beginning of the simulation, and this may be the result of the protein being affected by the ligand and interacting with the water molecules in the environment. The RMSD value of the system subsequently became stable at around 30 ns, indicating that the complex formed by the protein and ligand was stable in a solvent system (Fig. 9A).

In addition, the determination of the local structural flexibility of LasI*_Ha_* in the solo state and after binding to TF3 was performed by plotting the average fluctuations of all residues as the root mean square fluctuations (RMSF). The RMSF values of the residues in the ligand-binding region (two red boxes) were significantly smaller than those of the residues in the other regions (Fig. 9B), indicating that after the binding of the ligand to the active pocket of the protein, the ligand interacted significantly with the surrounding residues of the protein, consequently reducing the flexibility of these residues and further decreasing the overall flexibility of the protein. Lower flexibility may be more conducive to more stable substrate binding.

Hydrogen bonds play an important stabilizing role in noncovalent interactions between proteins and ligands (50). The result we obtained indicated that at the beginning of the simulation, the number of hydrogen bonds between LasI*_Ha_* and TF3 fluctuated significantly until the system reached 30 ns. By that time, the number of these hydrogen bonds became stable, indicating that a stable conformation was attained for the TF3-LasI*_Ha_* complex (Fig. 9C).

Changes in the secondary structure of the whole protein during the simulation were probed with the STRIDE algorithm to obtain the secondary structure with the most significant deviation during the simulation under known external stress (51). In such analysis, as the protein is subjected to additional pressure over the simulation, different color bands can be observed, which represent changes occurring in the secondary structure of the protein. In the case of LasI*_Ha_*, following TF3 binding, the α helix and β fold in the protein structure increased (Fig. 9D), indicating that the binding of a ligand to the protein could enhance the stability of the overall structure of the protein.

In addition, the longitudinal perspective on the binding system of small molecules to proteins and the results of different indicators throughout the simulation revealed a stable dynamic simulation process and so was the binding of small molecules to proteins, and no small molecule was found to detach from the protein (Fig. 9E), which also structurally explained the effectiveness of the LasI*_Ha_*-TF3 complex in being able to exert a sustained inhibitory effect.

### Conclusion

In this study, a highly active QSI against the QS system of *H. alvei* H4 was obtained by a virtual screening method based on the molecular docking technique. The obtained QSI, TF3, is found in black tea, and it is regarded as the most effective bioactive compound among the various TFs identified in tea. At sub-MIC concentrations, TF3 reduced the synthesis of AHLs and led to a remarkable down-regulation of QS gene expression and inhibition of bacterial phenotypes. The high anti-QS activity of TF3 stemmed from its strong interaction with LasI*_Ha_* to form a stable TF3-LasI*_Ha_* complex. Therefore, TF3 could be considered a potential QSI with excellent antimicrobial properties, and it could act as a base compound for the development of novel QSIs and their application in food preservation.
